# TMV mutants with poly(A) tracts of different lengths demonstrate structural variations in 3′UTR affecting viral RNAs accumulation and symptom expression

**DOI:** 10.1038/srep18412

**Published:** 2015-12-18

**Authors:** Song Guo, Elzbieta Kierzek, Gang Chen, Yi-Jun Zhou, Sek-Man Wong

**Affiliations:** 1 Department of Biological Sciences, National University of Singapore, Republic of Singapore; 2 Institute of Bioorganic Chemistry Polish Academy of Sciences, 61-704 Poznan, Noskowskiego 12/14, Poland; 3 Division of Chemistry and Biological Chemistry, School of Physical and Mathematical Sciences, Nanyang Technological University, 21 Nanyang Link, Singapore 637371; 4 Institute of Plant Protection, Jiangsu Academy of Agricultural Sciences; Jiangsu Technical Service Center of Diagnosis and Detection for Plant Virus Diseases, Nanjing 210014, PRC; 5 Temasek Life Sciences Laboratory, Singapore, Republic of Singapore; 6 National University of Singapore Research Institute in Suzhou, Jiangsu, PRC

## Abstract

The upstream pseudoknots domain (UPD) of *Tobacco mosaic virus* (TMV) is located at the 3′-untranslated region (UTR). It plays an important role in virus replication and translation. To determine the importance of UPD and 3′-UTR, and the effects of introduced RNA elements in TMV 3′-UTR, a series of TMV mutants with internal poly(A) tract upstream of UPD was constructed for structural analysis by selective 2′-hydroxyl acylation analyzed by primer extension (SHAPE). TMV(24A+UPD) and TMV(42A+UPD) formed a similar structure as that of TMV 3′-UTR, but TMV(62A+UPD) structures altered by the introduced poly(A) tract. In addition, TMV(24A+UPD) had a higher viral RNAs accumulation than TMV in *N. benthamiana* protoplasts, and induced lethal symptoms in the infected plants. TMV(62A+UPD) showed a drastically reduced accumulation, its coat protein was undetectable in protoplasts, and the inoculated plants remained symptomless. This study analyzed the structures of 3′-UTR of TMV and found that the longer poly(A) tract introduced upstream of UPD reduced viral RNAs accumulation and induced milder symptoms in *N. benthamiana*. In conclusion, different lengths of the internal poly(A) tract introduced into the TMV 3′UTR lead to structural variations that affect virus accumulation and symptom expression.

*Tobacco mosaic virus* (TMV) is a positive-sense single-stranded RNA virus that belongs to the genus *Tobamovirus*. TMV has a wide host range and the symptoms vary with plant species and environmental conditions. In the field, tobacco plants infected by TMV show a typical mosaic pattern of light and dark green colour on the leaves, and distortion of newly emerged leaves. In the greenhouse, TMV infected *N. benthamiana* plants show severe leaf necrosis and stem collapse, leading to plant death within a few days. Similar to other tobamoviruses, the genome of the TMV-U1 strain^1^,^2^ encodes four major proteins, two RNA-dependent RNA polymerases (p126/p183), a movement protein (p30) and a coat protein (p17.5), which are essential for virus replication, cell-to-cell and long distance movement in plants^3^,^4^,^5^, respectively. A poly(CAA) segment of nucleotides located in the 5′ untranslated region (UTR) serves as a binding site for the host heat shock protein, HSP101, which is essential for viral translation. The poly(CAA) sequence is functionally similar to a 5′-cap or a poly(A) tail that recruits host elongation factor eIF4F for viral translation^6^,^7^,^8^. In TMV 3′-UTR, five pseudoknots (PKs) are present. Three belong to an upstream pseudoknots domain (UPD), and two are located in the tRNA-like structure (TLS)^9^,^10^. TMV UPD plays an important role in both viral replication and translation. Mutational analysis shows that UPD could interact with eEF1A/GTP with high affinity, and the PK2 and PK3 of the UPD located immediately upstream of the TLS are involved^11^,^12^.

Polyadenylation is known to function in regulating RNA stability and translation. The importance of the poly(A) tail to viral infectivity has been reported in some viruses and the poly(A) length could also affect virus replication^13^,^14^,^15^,^16^. A poly(A) tail of sufficient length in *Bamboo mosaic virus* interacts with secondary structural elements upstream to form a pseudoknot structure, and disruption of this interaction reduces virus accumulation^17^,^18^. Several plant viral RNAs do not have a poly(A) tail, but possess an internal polyadenylate sequence in the 3′ terminal region of their genomes^19^. The length of the poly(A) tract varies in different viruses. So far, internal poly(A) tracts are found in bromoviruses, hordeiviruses, and tymoviruses^20^*. Dulcamara mottle virus* possesses an internal poly(A) tract in the 3′-UTR which lacks a 3′-terminal tRNA-like sequence (TLS). This is different from other tymoviruses^21^.

In recent years, two tobamoviruses isolated from Hibiscus spp. were reported to contain an internal poly(A) tract upstream of 3′-TLS. They are *Hibiscus latent Singapore virus* (HLSV)^22^ and *Hibiscus latent Fort Pierce virus*^23^,^24^. The length of HLSV internal poly(A) tract ranges from 78 to 96 nt^22^. HLSV is unable to replicate when the length of internal poly(A) tract is less than 24 nt^25^. Therefore, the 24 nt internal poly(A) tract is the minimal length required for HLSV replication in *N. benthamiana.* Moreover, HLSV is unable to replicate if the internal poly(A) tract is substituted by TMV UPD sequences, indicating that the internal poly(A) tract is not replaceable. This is different from previous studies that the UPD can functionally substitute for a poly(A) tail in plant and animal cells^26^.

In general, the 3′-UTR is considered as a modular element that largely provides self-contained functions. The TLS mimics the tRNA structure, and it enhances virus translation and promotes minus-strand synthesis^27^,^28^. *In vitro* competition experiments reveal that the TMV-TLS has the highest binding affinity for RNA polymerase^29^. Deletion studies of UPD showed that the pseudoknot which is closest to the TLS is essential for TMV replication^11^. It is known that the pseudoknot structure, not sequences in UPD, plays an important role in virus replication. The poly(A) tract is a simple RNA element and its length affects virus replication efficiency of HLSV. In this study, a series of TMV mutants were constructed with different lengths of internal poly(A) tract. The internal poly(A) tract is positioned upstream of UPD in the TMV genome. Results showed that TMV(24A+UPD) possessed the highest infectivity and induced most severe lethal symptoms. The accumulation of viral RNAs induced by TMV(24A+UPD) was higher than that of TMV in *N. benthamiana*. TMV(42A+UPD) induced necrosis in the inoculated leaves, and systemic movement to the upper leaves was slower than that of TMV(24A+UPD). The accumulation of viral RNAs of TMV(62A+UPD) declined rapidly in *N. benthamiana* protoplasts and infected plants were symptomless after inoculation. The RNA structures of the mutated TMV fragments were subjected to selective 2′-hydroxyl acylation analyzed by the primer extension (SHAPE) method. Structural change in the 3′-UTR was dependent on the length of the poly(A) tract that was introduced upstream of the UPD in TMV genomes, and the longer the tract, the lower the viral RNAs accumulation and its infectivity.

## Results

### TMV(24A+UPD) replicated in *N. benthamiana* and induced rapid plant death

**Figure 1:**
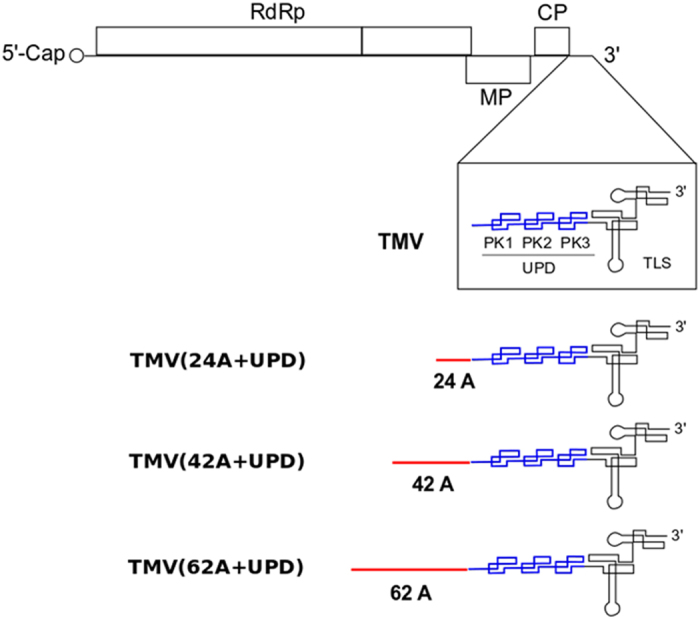
Schematic structure of TMV and its mutants with different lengths of internal poly(A) tract (24A, 42A and 62A) introduced before the upstream pseudoknotted domain (UPD). The genome of TMV contains a 5′ cap and a 3′ tRNA-like structure (TLS) with open reading frames of RNA-dependent RNA polymerase (RdRp), movement protein (MP) and coat protein (CP).

**Figure 2:**
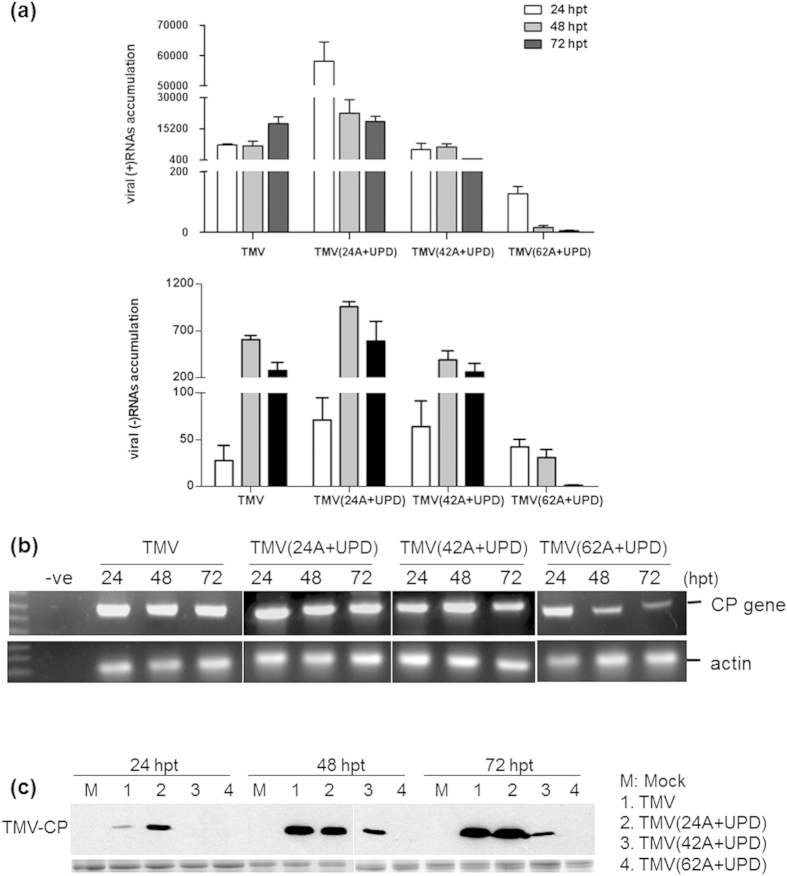
(**a**) Accumulation of TMV and its mutants positive and negative strand viral RNAs in transfected *Nicotiana benthamiana* protoplasts at 24, 48 and 72 hpt, respectively. Figure 2a represents the results of qRT-PCR. (**b**) RNA accumulation level and (**c**) coat protein expression of TMV and its mutants as determined by a regular RT-PCR and Western blot in transfected *N. benthamiana* protoplasts at 24, 48 and 72 hpt, respectively. The CP gene accumulation of TMV(62A+UPD) was reduced over time and its CP expression was not detected.

**Figure 3:**
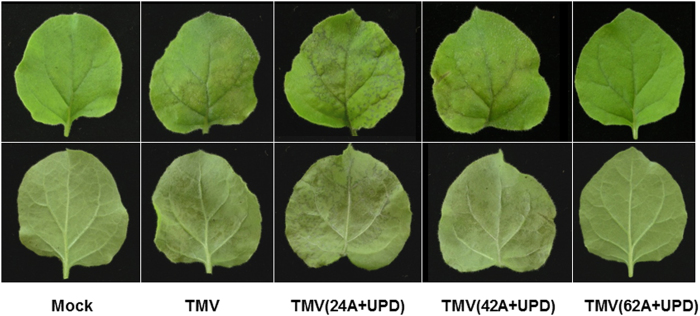
Close-up of TMV and its mutants inoculated leaves at 5 dpi. Upper and lower rows represent the adaxial and abaxial of the same leaves, respectively. TMV(24A+UPD)-inoculated leaf showed water-soaked lesions and more extensive necrotic patches than TMV and TMV(42A+UPD). TMV(62A+UPD) and mock inoculated leaves showed no symptoms.

**Figure 4:**
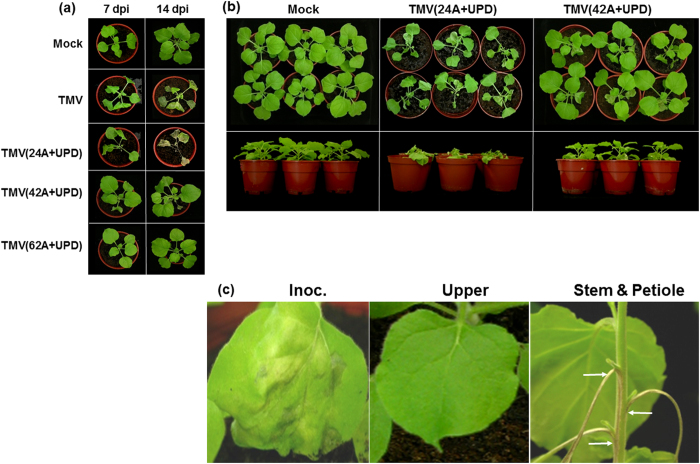
(**a**) Symptoms of *Nicotiana benthamiana* plants infected with TMV and its mutants at 7 and 14 dpi, respectively. (**b**) Side view of the infected plants inoculated with TMV(24A+UPD) and TMV(42A+UPD) at 7 dpi. (**c**) Close-up of TMV(42A+UPD)-inoculated plants at 7 dpi, showing necrotic lesions on the inoculated leaf, symptomless on the upper leaf and necrosis on stem and petiole.

TMV(24A+UPD) contained an internal poly(A) tract of 24 nt which was introduced upstream of TMV UPD at the 3′-UTR (Fig. 1). It replicated in *N. benthamiana* protoplasts and the accumulation of CP was higher than that of TMV in transfected protoplasts (Fig. 2). *N. benthamiana* plants inoculated with *in vitro* transcripts of TMV(24A+UPD) showed water-soaked lesions on the inoculated leaves and developed severe necrosis 5 days post inoculation (dpi) (Figs 3 and 4). The stem necrosis observed on TMV(24A+UPD)-inoculated plant leaves occurred earlier (4 dpi) than that on TMV-infected plants (6 dpi), resulting in a more rapid plant death than plants infected with TMV. Thus, the insertion of a 24 nt internal poly(A) tract upstream of the UPD in the TMV genome results in a higher infectivity and more severe necrosis in the inoculated plants.

### TMV(42A+UPD) systemic infection in *N. benthamiana* was slower than that of TMV(24A+UPD)

In HLSV, longer internal poly(A) tracts increase viral infectivity^25^. Therefore, it is reasonable to assume that the same effect would also occur in TMV genome. Two mutant viruses, TMV(42A+UPD) and TMV(62A+UPD), were constructed with an introduced poly(A) tract that was longer than 24 nt (Fig. 1). However, the longer poly(A) tract did not increase TMV infectivity. The viral RNAs accumulation of TMV(42A+UPD) and TMV(62A+UPD) were much lower than that of TMV(24A+UPD) in the transfected *N. benthamiana* protoplasts. Although the coat protein of TMV(42A+UPD) could be detected after 48 hours post transfection (hpt) in transfected protoplasts, its expression level was lower than that of TMV(24A+UPD). Unlike TMV(24A+UPD) which produced lethal symptoms in *N. benthamiana*, TMV(42A+UPD) produced necrotic lesions on the inoculated leaves at 5 dpi, but the upper leaves showed no symptoms (Figs 3 and 4). At 7 dpi, severe necrosis appeared on the stem and petioles of the infected plants, but the upper leaves remained symptomless (Fig. 4c).

TMV(24A+UPD) infection caused lethal symptoms with a higher accumulation level of viral RNAs in the upper leaves. However, after introducing a longer poly(A) tract, the accumulation of viral RNAs was significantly reduced in the upper leaves of TMV(42A+UPD)-infected plants. In order to test whether TMV(42A+UPD) systemic movement was inhibited by the introduced longer poly(A) tract of 42 nt, we compared the viral RNAs accumulation in both inoculated and upper leaves from TMV(24A+UPD)- and TMV(42A+UPD)-inoculated plants. As TMV(24A+UPD) replicates faster than TMV(42A+UPD) in protoplasts, we tested two different amounts of viral RNA transcripts (250 ng and 2.5 μg, respectively) to compare viral RNAs accumulation of TMV(42A+UPD) and TMV(24A+UPD).

**Figure 5:**
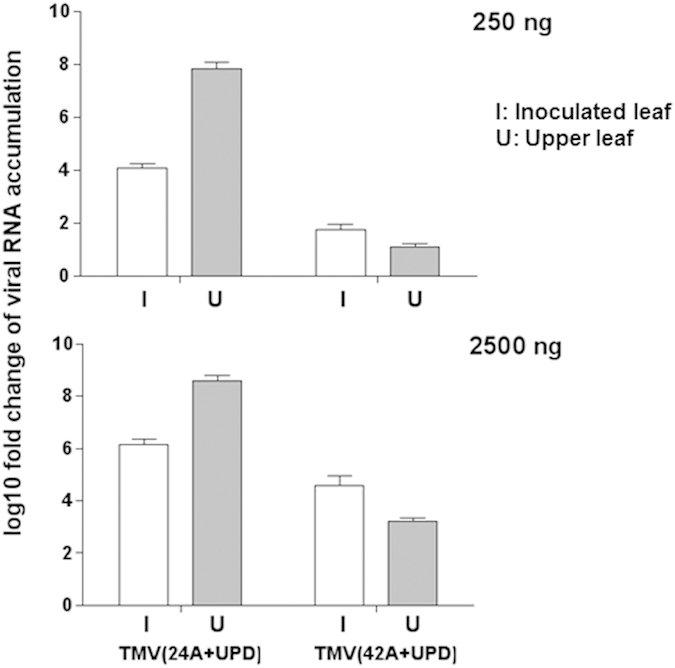
RNA accumulation of TMV(24A+UPD) and TMV(42A+UPD) in the inoculated and upper leaves using two different inoculum amounts (250 ng and 2500 ng, respectively), as determined by qRT-PCR at 6 dpi. Relative fold-change to *actin* gene in plants. Three biological replicates and each biological replicate contain three technical replicates.

Using an inoculum of either 250 ng or 2.5 μg, viral RNAs accumulation of TMV(42A+UPD) in the upper leaves was lower than that of TMV(24A+UPD). The viral RNAs accumulation of TMV(42A+UPD) in the upper leaves increased by using 2.5 μg inoculum compared with that of using 250 ng. Despite using ten times the amount of TMV(42A+UPD) as inoculum, the viral RNAs accumulation in the upper leaves was still significantly lower than that of TMV(24A+UPD) (Fig. 5). In addition, comparing the symptoms induced in *N. benthamiana* plants at 7 dpi, the side view of TMV(24A+UPD) infected plants (Fig. 4b) clearly showed the whole plant collapsed as a result of severe tissue necrosis. However, TMV(42A+UPD) inoculated plants survived, their upper leaves remained symptomless, and stem necrosis was restricted only to the internodal regions (Fig. 4c).

### TMV(62A+UPD) was unable to accumulate in *N. benthamiana*

TMV(62A+UPD)-inoculated plants were symptomless at 7 dpi (Figs 3 and 4a). Virus accumulation of TMV(62A+UPD) in *N. benthamiana* protoplasts at 24, 48 and 72 hpt was also monitored. The qRT-PCR results showed that the accumulation of TMV(62A+UPD) genomic RNA did not increase, in comparison to TMV and two other mutants (Fig. 2a). Although the negative strand viral RNAs could be detected in the transfected protoplasts, the RNA level was decreasing over time. The CP gene expression of TMV(62A+UPD) declined noticeably in the transfected protoplasts over time (Fig. 2b). No CP was detected in transfected protoplasts after 72 hpt (Fig. 2c). These results indicated that TMV(62A+UPD) CP translation was interrupted by the introduced 62 nt internal poly(A) tract.

### SHAPE-probed secondary structure analyses of RNA fragments in TMV-3′-UTR and its mutants

**Figure 6:**
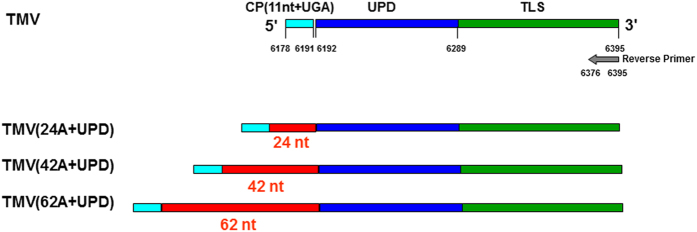
Schematic sequences used for SHAPE-probed structural analyses. TMV sequence starts from 6178 nt to the 3′-end (6395 nt), consisting of 11 nucleotides and UGA stop codon of TMV-CP gene, UPD and TLS. The mutants were added with 24 nt, 42 nt and 62 nt internal poly(A) tracts, respectively, downstream of the CP gene UGA stop codon.

Since there is no internal poly(A) tract present in the TMV genome, we propose that the introduced internal poly(A) tract in the TMV mutants would remodel the RNA structure of the 3′-UTR. The structures of the TMV 3′-UTR fragment and its mutants were analyzed by the SHAPE probing method (Fig. 6). For TMV, the sequence was taken from 6178 to 6195 nt of the full-length viral genome which contains the last 11 nt of the TMV-CP gene and the stop codon UGA, and TMV-3′-UTR nucleotides. For the mutants, the sequences consisted of the same region of the TMV-CP gene but with insertions of internal poly(A) tract of 24 nt, 42 nt and 62 nt, respectively. The primer used for reverse transcription targeted the 3′-end of TMV genome from 6376 to 6395 nt. The three consecutive pseudoknots^30^ that consists of UPD were labeled separately to show nucleotides reactivity changes after the introducing of poly(A) tract.

**Figure 7:**
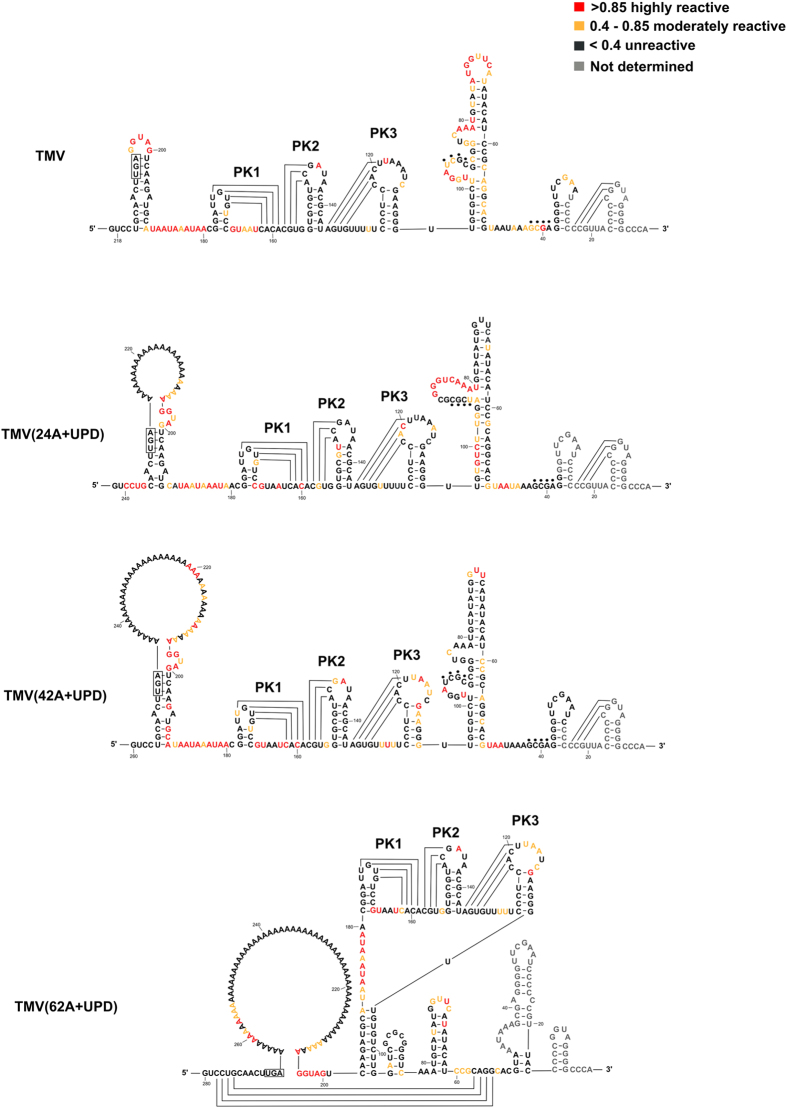
SHAPE analysis of 3′-terminal structure of TMV and its mutants. Black dots represent base pairings between CGCU and GCGA.

In the structure of TMV(24A+UPD) (Fig. 7), the introduced poly(A) tract enlarged the apical loop in stem-loop structure which located at the 5′-end of the sequence. Also, the basal stem in the stem-loop structure became two base pairs less than that of TMV. The PK1, PK2 and PK3 structures were same as that of TMV. The bulge in the longer stem-loop structure that downstream of UPD was different from TMV and SHAPE values of nucleotides G_97_UUCUGU_101_ turned to be reactivate in TMV(24A+UPD). In TMV(42A+UPD) structure (Fig. 7), the apical loop became larger as the length of poly(A) tract increased. The nucleotides A_190_C_200_G_201_ that used to form the basal stem became reactivate. However, the PK1, PK2 and PK3 structures were the same as that of TMV.

The structure of TMV(62A+UPD) (Fig. 7) was different from those of TMV, TMV(24A+UPD) and TMV(42A+UPD). The introduced internal poly(A) tract formed a larger loop upstream of UPD and TLS sequences. Although the pseudoknots formed by PK1, PK2 and PK3 sequences were identical to that of TMV, the structure downstream of UPD (TLS region) was altered compared to that of TMV. The SHAPE reactivity in the TLS region of TMV(62A+UPD) is lower than TMV (Supplementary Fig. 1). The lack of SHAPE reactivity would due to the increased conformational dynamics that caused by the presence of the introduced 62 nt poly(A) sequence.

The SHAPE analysis of those TMV poly(A) tract mutants showed that the stability of PK1, PK2 and PK3 was not greatly affected by the introduced internal poly(A) tracts of different lengths. Besides the large internal loop contributed by the internal poly(A) tracts, the obvious alteration in TMV(62A+UPD) was the disruption of the stem-loop that originally located at the 5′-end of TMV sequence. Also, the structure of TLS in TMV(62A+UPD) was altered. The TLS was proven to be important for TMV replication^10^. The structural variations in TMV(62A+UPD) could affect virus replication and accumulation. Moreover, the TMV-CP stop codon UGA could form base pairing in both TMV, TMV(24A+UPD) and TMV(42A+UPD). But in TMV(62A+UPD), such base pairing was destabilized due to the presence of a larger loop that formed mainly with 62 nt internal poly(A) tract. Taken together, the introduced internal poly(A) tract of 62 nt resulted in altered 3′-UTR structure, which may account for the non-infectivity of TMV(62A+UPD).

The SHAPE values of each nucleotide in TMV sequence and its mutants were listed in bar chart to compare the reactivity changes after introducing internal poly(A) tract (Supplementary Fig. 1). Compared with that of TMV, the reactivity of nucleotides in TLS sequence decreases along with the internal poly(A) tract length elongation. It was also noticed that some adenine residues in the internal poly(A) tract were unreactive, which may be due to the fact that poly(A) tract can form a single helix structure with the ribose sugars in C3′-endo conformation^31^ resulting in no SHAPE reactivity. It is also possible that these adenine residues might be involved in the interactions with the other parts of the structure, and thus affected the original structure and functions of the RNA. The long internal poly(A) tract in TMV(42A+UPD) might block part of UPD region in binding to proteins such as eEF1A/GTP^11^,^12^. In TMV(62A+UPD) structure, the internal poly(A) tract in a helical conformation remodeled the structure and varied the dynamics of UPD and TLS.

## Discussion

Attempts have been made to predict the secondary structure of TMV 3′-UTR using a variety of chemical and enzymatic tools^9^,^32^. SHAPE combines a novel chemical probing technology with reverse transcription, capillary electrophoresis, and secondary structure prediction software to rapidly determine the structure of RNAs in a single-nucleotide resolution. It is particularly useful in predicting RNA secondary and tertiary interactions^33^,^34^,^35^. We applied the SHAPE approach to gain insight into TMV 3′-UTR conformation. This methodology employs an electrophilic reagent that reacts selectively with the 2′-hydroxyl ribose group of single-stranded nucleotides to create a covalent 2′*-O-ribose* adduct, while the 2′-hydroxyl group of structurally constrained residues shows reduced nucleophilic reactivity. In contrast to conventional chemical and enzymatic probing techniques, SHAPE provides structural information at every nucleotide position^36^.

In this study, a series of mutants demonstrated the importance of secondary structures within the 3′-UTR of TMV genome. After introducing a 24 nt internal poly(A) tract upstream of UPD in the TMV genome, we found that the altered virus killed the plants more rapidly than the wild-type TMV, indicating that an introduced internal poly(A) tract could enhance viral virulence. However, after increasing the introduced internal poly(A) length to 62 nt, there was no symptom observed on TMV(62A+UPD)-inoculated plants, and this mutant virus showed a rapidly diminishing accumulation of viral RNAs in transfected *N. benthamiana* protoplasts over time (Fig. 2). These results showed that the length of introduced internal poly(A) tract affect viral RNAs accumulation and symptom expression. The SHAPE analysis confirmed that such effects result from RNA structural variations in the 3′-UTR.

The effects of poly(A) tail length on viral replication have been reported in *Coronavirus*^37^,^38^. Its 3′ poly(A) tail lengths of genomic and subgenomic mRNAs were extended during infection. Moreover, functional analyses revealed that CP accumulation of *Coronavirus* with a longer poly(A) tail was enhanced. This result is contrary to our result in that the mutant viruses with a longer internal poly(A) tract showed reduced replication in *N. benthamiana* protoplasts and less virulence in the whole plants (Figs 2 and 4). The difference between a poly(A) tail and an internal poly(A) tract lies in its relative position in the viral genome. In the mutants, both UPD+TLS are located downstream of the internal poly(A) tract. The TMV-TLS has been known to function as a 3′-translational enhancer, which plays a similar role of stabilizing the viral RNA genome. The longer the poly(A) tail, the more stable is the virus. However, the internal poly(A) tract is located upstream of UPD+TLS, not at the 3′ terminus of its genome. The structure formed by the internal poly(A) tract could affect normal functions of UPD+TLS, as our results have shown. When longer internal poly(A) tracts were introduced into TMV genome, accumulation level of the mutant viruses decreased (Fig. 2).

We have chosen 24 nt to be inserted into TMV genome (TMV-24A+UPD) because 24 nt internal poly(A) tract is the minimal length for HLSV replication^25^. As previous results also showed that longer internal poly(A) tract in HLSV enhanced virus replication^25^, we then decided to insert 44 A and 64 A for the internal poly(A) tracts for testing. However, after several attempts, we only managed to obtain inserts of variable lengths and we settled on closest lengths of 42A and 62A. The length variation is probably due to polymerase slippage during PCR. To our surprise, the longer poly(A) tract introduced upstream of UPD did not enhance virus replication, as in the case for HLSV. SHAPE analysis revealed that the TMV mutants of different internal poly(A) tracts are correlated to structural alterations in the 3′-UTR of viral genome.

Before introducing the internal poly(A) tract to TMV genome, we tested whether the UPD of TMV could be replaced with an internal poly(A) tract. TMV(43A) has the UPD replaced by an internal poly(A) tract of 43 nt^25^ and it could replicate in *N. benthamiana* protoplasts. However, the replication and accumulation of TMV(43A) were significantly reduced, as compared with that of TMV. Also, different from the lethal symptom caused by TMV infection, TMV(43A) induced a mosaic symptom and its infected plants remained alive after infection. These results indicated that the UPD of TMV alone could be replaced with an internal poly(A) tract but with a reduced replication efficiency.

The poly(A) tract of HLSV is located upstream of TLS and longer poly(A) tract upstream of 3′-TLS would result in higher accumulation of the viral RNA^25^, which is contrary to the observations made in this study. In our TMV mutants, as the UPD and 3′-TLS in TMV genome were inseparable, the poly(A) tract was introduced upstream of the UPD, instead of TLS. It has been reported that TMV could not replicate if its entire UPD was deleted or when the pseudo knot located at the nearest upstream of the 3′-TLS was altered or deleted^11^. In addition, the reduced replication of TMV(43A) also supports the hypothesis that UPD and 3′-TLS in TMV genome function cooperatively^25^,^26^,^39^. As we aimed to test the effects of the length of an internal poly(A) tract on TMV replication, the UPD and 3′-TLS were kept intact in order to prevent disruption of UPD and 3′-TLS, as mutations between the UPD adjacent to 3′-TLS would abolish TMV replication.

TMV(24A+UPD) replicated more rapidly than TMV in *N. benthamiana.* The internal poly(A) tracts of 24 nt, 42 nt and 62 nt caused symptoms of rapid plant death, localized tissue necrosis, and symptomless, respectively. TMV(42A+UPD) induced necrosis only in the inoculated leaves and petioles, and no symptoms were observed in the upper leaves at 14 dpi. Additionally, in the crude sap of TMV(42A+UPD)-infected leaves, virus particles were observed as flexuous, rod-shaped structures after negative staining, indicating that the virus could assemble with a 42 nt internal poly(A) tract (Supplementary Fig. 2). TMV(42A+UPD) showed a similar structure as that of TMV, but the terminal loop in the stem-loop that located close to the 5′-end sequence was enlarged. The poly(A) tract in TMV(42A+UPD) may interact with UPD which affects virus replication by blocking the UPD interactions with host factors, resulting a decreased viral RNAs accumulation and negatively affect virus systemic movement.

According to the SHAPE-probed structural analysis, TMV(62A+UPD) 3′-terminal structures were distinct from that of TMV. TMV(62A+UPD) showed a reduced viral RNAs expression level and its CP was undetectable in the transfected *N. benthamiana* protoplasts. The absence of CP suggests that translation of viral proteins may be inhibited. Since there was no CP expression, encapsulation was not possible, resulting in degradation of TMV(62A+UPD) viral RNAs in the cytoplasm. The structural analysis of TMV(62A+UPD) showed that the conformation of the original TMV TLS was altered. As TLS is important for TMV replication by enhancing viral RNAs stability during translation, the altered TLS in TMV(62A+UPD) could be the reason contributing to the lack of CP expression. The larger hairpin loop of the 62 nt poly(A) tract may lead to the absence of CP expression and symptom expression. Most of the *cis*-acting RNA elements in RNA viruses are local sequences or confined secondary or tertiary structures^40^,^41^,^42^,^43^. The 5′- and 3′-UTRs of TMV interact with each other to form a closed loop that initiates virus translation. There is increasing evidence that long-range RNA base paring interactions also have essential functions, such as executing and modulating various viral processes^44^,^45^,^46^,^47^. Therefore, the large hairpin loop in TMV(62A+UPD) may pose steric hindrance to the region of interaction, or binding to several nucleotides complementary to the 5′-terminal sequences, preventing the long-range RNA-RNA interaction.

Since the large hairpin loop was located upstream of the pseudoknot domain, interaction between the UPD and host eEF1A may also be affected by the structural alteration. Although the negative- strand of TMV(62A+UPD) viral RNAs were detected from the transfected protoplasts by qRT-PCR, it showed a decreasing trend and the amount was significantly lower than that of TMV, TMV(24A+UPD) and TMV(42A+UPD). The altered RNA structure of TMV(62A+UPD) might affect viral translation efficiency, so the amount of RNA-dependent RNA polymerase would be reduced, leading to a reduced production of negative-sense viral RNA templates.

In this study, instead of the entire genome sequences, the 3′-terminal 14 nucleotides of the CP ORF plus the 3′-UTR region of TMV and its mutants were used for SHAPE analysis. As the long range interaction of TMV 5′- and 3′-UTRs is mediated through host proteins, instead of direct RNA-RNA interaction^42^,^47^,^48^,^49^, using full-length RNA for SHAPE analysis would not reflect the long range interaction. The current SHAPE analysis applied has taken rational design that included a sufficiently long region of RNA (about 300 nt) which covers the RNA region of interest, internal poly(A) tract, UPD and TLS sequences. Also, the primer binding at the 3′-end of TLS sequence would have less effects on the RNA conformations of UPD and internal poly(A) tract. At the 5′-end of the RNA sequence, there was a short sequence consisting of 14 nucleotides (11nt + UGA) located upstream of the internal poly(A) tract for SHAPE analysis. It is likely that the structure of upstream regions may also be altered. Currently, the results obtained from using the partial sequence of TMV and the mutants have clearly demonstrated the structural differences in the 3′-UTR. This is a widely accepted strategy used to analyze a local RNA region of interest^50^,^51^,^52^,^53^. In addition, mapping of long RNA using a whole viral genome^54^ is not common, as technically it is not trivial to achieve desirable results. Potentially it can lead to mapping of mis-folded structure if the RNA is folded without the appropriate interacting proteins involved. Also, there is a high possibility of having not only one but a mixture of RNA structures and it is difficult to detect and to avoid. It is more straight-forward to verify the folding of 300 nt RNA on native gels to establish appropriate conditions of folding leading to a single structure for analysis. Therefore, we choose RNA sequence covering partial CP ORF and the 3′-UTR region of TMV and its mutants for the SHAPE analysis. Results showed that the length of introduced poly(A) tract could affect virus replication/accumulation and symptoms induced in *N. benthamiana*. Additionally, in order to monitor the variation of introduced poly(A) tract in TMV genome, we also verified the genomic sequence of TMV(24A+UPD) after its replication in *N. benthamiana*. The result showed that the internal poly(A) tract was also elongated as shown in HLSV. As our results showed that longer poly(A) tract upstream of UPD leads to reduced TMV replication, the elongation of internal poly(A) tract is likely to result from polymerase slippage during virus replication and it is an inherent property that exist in both HLSV and TMV mutants.

In conclusion, this study analysed the 3′-UTR structure of TMV with introduced internal poly(A) tracts of different lengths through SHAPE analysis. It revealed that the longer the introduced poly(A) tract, the lower the virulence of the mutants. This appears to be associated with structural variations and dynamic changes. This research work represents the first application of SHAPE analysis to TMV 3′-UTR and comparison of the length effect of introduced internal poly(A) tracts on viral accumulation. Our results provide a better understanding of the functions of UPD+TLS in relation to RNA structures in TMV. In addition, the new knowledge on the effects of different internal poly(A) tract lengths on virus accumulation has potential application in engineering TMV and its mutants into high-efficiency expression vectors.

## Materials and Methods

### Plasmid Construction

Using a TMV full-length cDNA clone as template, primers 24AUPD-F and 24AUPD-R were designed to introduce a 24 nt internal poly(A) tract upstream of its UPD. High fidelity enzyme (KAPA Biosystems) was used for over-lapping PCR. *DpnI* was added into the PCR products and incubated at 37 °C for 30 min to remove the original DNA template. The treated PCR products (5 μl) were transformed into competent *E. coli* cells by heat-shock treatment. Colonies were screened by sequencing the transformed plasmids on an Ampicillin-LB medium to confirm the introduced internal poly(A) length. The same approach was used to construct TMV(42A+UPD) and TMV(62A+UPD). The sequences of primers were listed in Table 1.

### *In vitro* transcription

Full-length cDNA clone of TMV and the mutants were linearized with *KpnI*. After phenol/chloroform extraction and ethanol precipitation, the purified DNAs were used as templates for *in vitro* transcription by the mMessage mMachine *in vitro* transcription kit (Life Technologies, Ambion). RNA transcripts were precipitated by LiCl, following the manual instructions and dissolved in RNase-free water.

### Plant inoculation and qRT-PCR

Fully expanded leaves of 4-week-old *N. benthamiana* were mechanically inoculated with 2 μg *in vitro* transcribed viral RNA in GKP buffer (50 mM glycine; 30 mM K_2_HPO_4_, pH 9.2; 1% bentonite; 1% celite). Total RNAs were extracted from the inoculated and upper leaves by TRIzol reagent. qRT-PCR was performed using the KAPA SYBR Fast qPCR kit on a CFX384 Real-Time PCR system (Bio-Rad). The *N. benthamiana actin* gene was used as an internal control. Error bars (SD) were obtained from nine replicates that included three biological replicates and each biological replicate contains three technical replicates. Primer-R and Primer-R-negative-strand were used for positive- and negative-strands reverse transcription, respectively. Primers qPCR-F and qPCR-R were used for target fragment amplification and primer Actin-F and Actin-R were used to amplify the *actin* gene. The sequences of primers were listed in Table 1.

### Protoplast transfection, total RNA isolation and Western blot

*Nicotiana benthamiana* protoplasts were isolated and transfected with *in vitro* transcripts of TMV and its mutants by polyethylene glycol transfection. Total RNAs were extracted from transfected protoplasts with TRIzol reagent at 24 h, 48 h and 72 h, respectively, followed by cDNA synthesis with primer R using the SuperScript III Reverse Transcriptase kit (Invitrogen). TMV-CP gene was amplified using primers TMV-CP-F and TMV-CP-R. *Actin* was used as internal control. Total proteins isolated from the transfected protoplasts at different time points were used for Western blot to detect CP expression using TMV-CP antibody.

### SHAPE structure probing

Primer TMV-T7(CP_14_+UPD)-F and primer R (Table 1) were used to amplify target fragments from TMV and Primer TMV-T7(CP_14_+AAA)-F and primer R (Table 1) were used for TMV mutants TMV(24A+UPD), TMV(42A+UPD) and TMV(62A+UPD) fragment amplification. From 5′ to 3′ orientation, the fragment consisted of 14 nt from the CP ORF 3′ end sequence, 24 nt upstream of UPD, UPD(PK1, PK2, PK3), and TLS in TMV genome. For the three mutants, the inserted 24 nt, 42 nt and 62 nt were located downstream of CP stop codon in the fragments (Fig. 6), respectively. The PCR products were column-purified by the Wizard^®^ SV Gel and PCR Clean-up System (Promega) and used as templates for *in vitro* transcription using AmpliScribe T7-Flash transcription kit. The RNA transcripts were purified by denaturing PAGE gel purification. After ethanol precipitation, RNA was dissolved in RNase-free water.

We used an experimental and signal processing pipeline that requires two fluorescent labels. Four reactions were set up for each sample. The four reactions were resolved in two capillaries: the (+) reagent reaction and a sequencing reaction were mixed for one capillary. Similarly, the (−) reagent reaction was mixed with an identical sequencing reaction.

Two *p*mol of RNA were mixed with RNA folding buffer (50 mM HEPES (pH 7.0); 100 mM KCl; 2.5 mM MgCl_2_ and denatured at 65 °C for 5 min. The RNA was then slowly cooled at room temperature for 30 min. Reactions were divided into two aliquots of 4.5 ul each and 0.5 ul NMIA (0.001 g in 125 ul DMSO) was added to one as the (+) reaction. Equal volume of DMSO was added to the other aliquot as the (−) reaction. Both (+) and (−) reactions were incubated at 37 °C for 40 min and precipitated at −20 °C with 60 ng/μl glycogen, 0.3 M sodium acetate, pH 5.2 and three volumes of cold ethanol. Precipitated RNA was collected by centrifugation, washed once in 70% ethanol and resuspended in RNase-free water. Two *p*mol of VIC or NED-labeled primer P6 were annealed to the RNA at 90 °C 3 min, 55 °C 10 min, on ice 3 min. RNA was reverse transcribed at 55 °C for 50 min with the Invitrogen superscript III transcriptase kit. Sequencing ladders were prepared using the cycle sequencing kit according to the manufacturer’s instructions and primers labeled with NED or VIC dyes which was different from that used for the (+) and (−) reactions. Sequencing reactions were divided into two and mixed with (+) and (−) reactions. RNA was precipitated as above, dried and resuspended in 15 μl Hi-Di formamide. Primer extension products were analysed on an Applied Biosystems 3100 xl DNA analyser. Electropherograms were processed using the QuSHAPE programme^55^, following the software developer’s protocol.

**Molecular modeling.**  The RNAstructure^56^ software package from the Mathews Lab (http://rna.urmc.rochester.edu/software.html) at University of Rochester Medical Center, was used to generate the secondary structures based on the 3′-UTR SHAPE-probed secondary structure information. The “Force-Read SHAPE Reactivity-Pseudo energy Constraints” option was chosen for the RNA modeling under default setting of Slope (kcal/mol) 1.8 and Intercept (kcal/mol) −0.6 with the analyzed sequence SHAPE file. The secondary structures and the corresponding SHAPES values were visualized using the VARNA^57^ Java applet and further drew by graphic software.

## Additional Information

**How to cite this article**: Guo, S. *et al.* TMV mutants with poly(A) tracts of different lengths demonstrate structural variations in 3'UTR affecting viral RNAs accumulation and symptom expression. *Sci. Rep.* **5**, 18412; doi: 10.1038/srep18412 (2015).

## Supplementary Material

Supplementary Information

## Figures and Tables

**Table 1:** List of primers used in this study.

Primer	Sequence (5′-3′)
Actin-F	CTTGAAACAGCAAAGACCAGC
Actin-R	GGAATCTCTCAGCACCAATGG
Primer-R	TGGGCCCCTACCGGGGGTAA
Primer-R-negative-strand	GTATTTTTACAACAATTACCAACA
TMV-T7(CP_14_+UPD)-F	ATTACA**TAATACGACTCACTATAGGG**GTCCTGCAACTTGAGGT
TMV-T7(CP_14_+AAA)-F	ATTACA**TAATACGACTCACTATAGGG**GTCCTGCAACTTGAAAAA
qPCR-F	AGGTGTACAGGTACAATGCG
qPCR-R	ACGAGTAGCATCTAACGTTT
Primer-R-NED	NED-TGGGCCCCTACCGGGGGTAA
Primer-R-VIC	VIC-TGGGCCCCTACCGGGGGTAA
24AUPD-F	AAAAAAAAAAAAAAAAAAAAAAAAGGTAGTCAAGATGCATAATAAATA
24AUPD-R	TTTT TTTTTTTTTT TTTTTTTTTTTCAAGTTGCA GGACCAGAGG TCCA
TMV-CP-F	ATGTCTTACAGTATCACTAC
TMV-CP-R	TCAAGTTGCAGGACCAGAGG
