# Supplementary material for: TMV mutants with poly(A) tracts of different lengths demonstrate structural variations in 3′UTR affecting viral RNAs accumulation and symptom expression
Source: Sci Rep. 2015 Dec 18;5:18412. doi: 10.1038/srep18412 (PMC4683447; doi:10.1038/srep18412)
Supplement: Supplementary Information [file srep18412-s1.pdf]

# Supplementary information

## Manuscript title:

## TMV mutants with poly(A) tracts of different lengths demonstrates structural variations in 3’UTR affecting viral RNAs accumulation and symptom expression

**Authors:**
**Song Guo, Elzbieta Kierzek, Gang Chen, Yi-Jun Zhou, Sek-Man Wong***

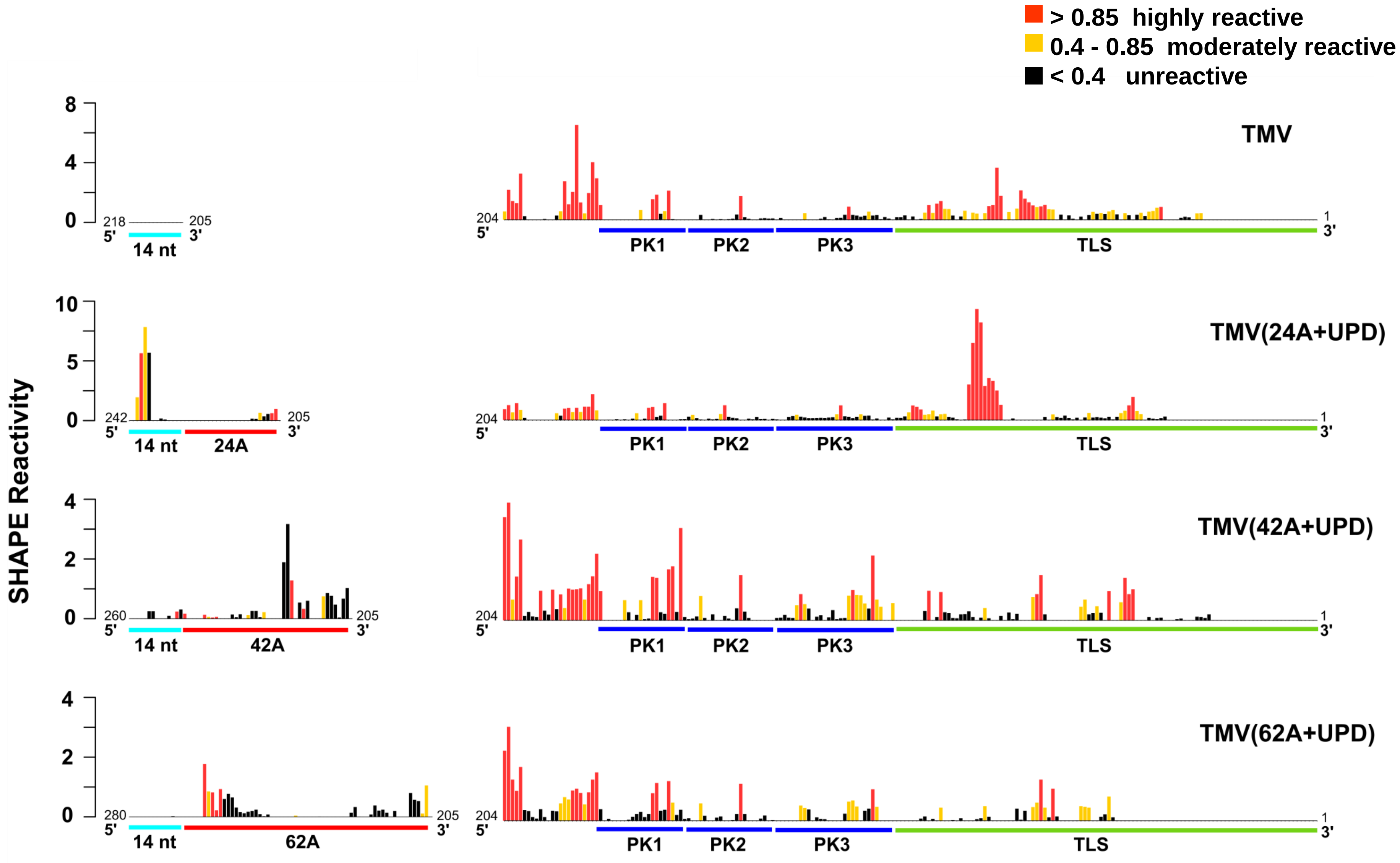


**Supplementary Figure 1. SHAPE reactivity of each nucleotide in TMV and its mutants sequences.**

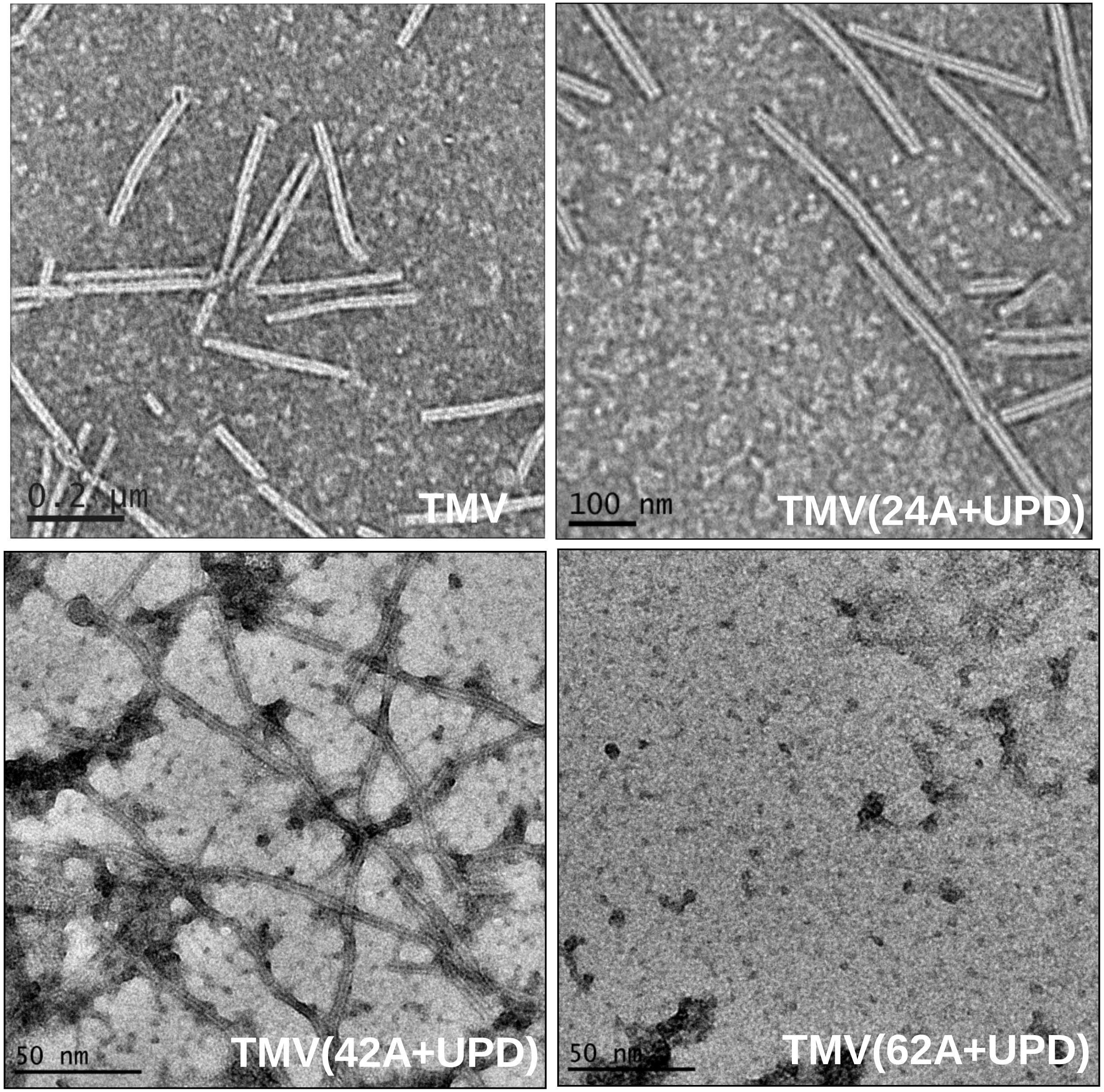


**Supplementary Figure 2. Transmission electron microscope (TEM) images of TMV and its mutants in the crude sap of inoculated *Nicotiana benthamiana* leaves at 5 dpi.**
